# Laser-Induced Graphene Electrodes Modified with a Molecularly Imprinted Polymer for Detection of Tetracycline in Milk and Meat

**DOI:** 10.3390/s22010269

**Published:** 2021-12-30

**Authors:** Biresaw D. Abera, Inmaculada Ortiz-Gómez, Bajramshahe Shkodra, Francisco J. Romero, Giuseppe Cantarella, Luisa Petti, Alfonso Salinas-Castillo, Paolo Lugli, Almudena Rivadeneyra

**Affiliations:** 1 Faculty of Science and Technology, Free University of Bolzano, 39100 Bolzano, Italy; Bajramshahe.Shkodra@natec.unibz.it (B.S.); giuseppe.cantarella@unibz.it (G.C.); paolo.lugli@unibz.it (P.L.); 2 Faculty of Chemical and Food Engineering, Bahir Dar Institute of Technology, Bahir Dar University, Bahir Dar 6000, Ethiopia; 3 Department of Analytical Chemistry, University of Granada, 18071 Granada, Spain; inmaog@ugr.es (I.O.-G.); alfonsos@ugr.es (A.S.-C.); 4 Pervasive Electronics Advanced Research Laboratory (PEARL), Department of Electronics and Computer Technology, University of Granada, 18071 Granada, Spain; franromero@ugr.es (F.J.R.); arivadeneyra@ugr.es (A.R.)

**Keywords:** laser-induced graphene, antibiotic residue, tetracycline, molecularly imprinted polymer, milk, meat, flexible, electrochemical sensor

## Abstract

Tetracycline (TC) is a widely known antibiotic used worldwide to treat animals. Its residues in animal-origin foods cause adverse health effects to consumers. Low-cost and real-time measuring systems of TC in food samples are, therefore, extremely needed. In this work, a three-electrode sensitive and label-free sensor was developed to detect TC residues from milk and meat extract samples, using CO_2_ laser-induced graphene (LIG) electrodes modified with gold nanoparticles (AuNPs) and a molecularly imprinted polymer (MIP) used as a synthetic biorecognition element. LIG was patterned on a polyimide (PI) substrate, reaching a minimum sheet resistance (R_sh_) of 17.27 ± 1.04 Ω/sq. The *o*-phenylenediamine (oPD) monomer and TC template were electropolymerized on the surface of the LIG working electrode to form the MIP. Surface morphology and electrochemical techniques were used to characterize the formation of LIG and to confirm each modification step. The sensitivity of the sensor was evaluated by differential pulse voltammetry (DPV), leading to a limit of detection (LOD) of 0.32 nM, 0.85 nM, and 0.80 nM in buffer, milk, and meat extract samples, respectively, with a working range of 5 nM to 500 nM and a linear response range between 10 nM to 300 nM. The sensor showed good LOD (0.32 nM), reproducibility, and stability, and it can be used as an alternative system to detect TC from animal-origin food products.

## 1. Introduction

Antibiotics are secondary metabolites of certain bacterial and fungal species; they are harmful to other bacterial species and can play crucial roles in agriculture, veterinary, and medicine [1,2,3]. Antibiotics are mainly employed to treat and prevent different diseases in animals such as gastrointestinal and respiratory diseases, arthritis, mastitis, and other bacterial infections [4]. Every year, around 63,150 tons of antibiotics are used worldwide as therapeutic, prophylactic, growth-promoting, and other kinds of treatments for animals [5]. Among these, tetracycline (TC) is one of the worldwide most used antibiotics to treat animals [6], since its discovery and introduction to the market in 1953 [7]. In particular, TC, oxytetracycline (OTC), chlortetracycline (CTC), and doxycycline (DC) are all grouped under the more general name of TC antibiotics [8] and are widely used in food-producing animals due to their low cost, availability, ease of administration, and broad-spectrum antimicrobial activity [9,10]. All TCs have a basic chemical structure consisting of a naphthacene core comprising four fused six-membered rings designated D, C, B, and A, to which a variety of functional groups are attached. They only differ from each other by the presence of chloride, methyl, and hydroxyl groups. The dimethylamino group at position C4 of the A ring has been shown to be necessary for antibacterial activity. TC, especially, is a heavily used antibacterial compound to treat bacteria-induced infections in animals. Excessive use and abuse of TC in food-producing animals can cause its accumulation in animal-origin foods such as milk, meat, and eggs [11]. As a consequence, its presence can affect consumers’ health, creating undesired allergic reactions, antimicrobial resistance, gastrointestinal disturbance, and TC pigmentation teeth [12]. Because of that, institutional organizations and health agencies have set maximum residue limits (MRL) of TC in foods, to protect consumer health. In particular, both the European Union (EU) and the World Health Organization (WHO) set 100 mg/L as the MRL, whereas the US Food and Drug Administration (FDA) set 80 mg/L as the safe level of TC residues in milk, and the Chinese government standard association (GB 18406.3-2001) [7] and EU [13] set 100 mg/kg of TC residues in meat as the MRL. Therefore, detection of antibiotic residues, such as TC, in animal products prior to consumption is a vital step to guarantee and protect consumers’ health. 

Many standard analytical methods such as high-performance liquid chromatography (HPLC) coupled to mass spectrometry (MS) or ultraviolet (UV) detection, capillary electrophoresis (CE), enzyme-linked immunosorbent assays (ELISA) [14], spectrophotometry, microbiological assays, and fluorescence and chemiluminescence detection [15] are used for the detection of TC residues in food. Among these, the ELISA method is one of the most commonly used screening tools due to its high sensitivity [16]. However, it has several disadvantages, including the need for an enzyme-labeled conjugate, the time-consuming production of the core reagent antibody (4–6 months), the nonreusable colorimetric detection method, the weak output signal, and the long measurement time required (2–4 h) [8]. Additionally, while ELISA and all the other methods mentioned above are sensitive and accurate, they all require expensive instruments, professional operators, complicated and time-consuming procedures for sample preparation, and multistep clean-up, unsuitable for on-site detection [17]. To overcome these limitations, developing rapid, label-free, sensitive, portable, and reliable techniques to detect TC residues in animal-origin foods is of crucial importance [18]. One possibility to realize such detection systems is to develop electrochemical sensors. Electrochemical sensors show unique advantages, such as a long stability or shelf-life, highly selectivity, reproducible sensing performance, tailorability/customizability, and rapid response [19]. MIP has unique advantages over natural biological receptors in terms of physical and chemical stability, ease of preparation, low cost, and applicability in harsh environmental conditions.

To date, electrochemical sensors have been realized using different recognition elements such as antibodies [20], enzymes [21], aptamers [22], and molecularly imprinted polymers (MIPs) [23]. Among the different recognition elements, MIP has become a promising technique for the functionalization of electrochemical sensors [24]. Molecular imprinting technology (MIT) is a technique to design artificial receptors with a predetermined selectivity and specificity for a given analyte, which can be used as ideal materials in various application fields. MIT has been widely used in separation, enrichment, and detection [25]. In fact, recently, MIP has been widely used as synthetic antibodies in electrochemical sensors due to their excellent selectivity, good physical and chemical stability, and low cost [26]. MIP forms a wide range of polymeric film for sensors, due to its simplicity and ease of preparation, stability, and reliability [27] and has been applied in sensors that mimic an antibody recognition due to its specific affinity and selectivity to the template [28,29]. MIP amalgamation techniques based on the nature of the chemical bonds involved can be classified into either a covalent approach in which a template–monomer complex is formed through reversible covalent or noncovalent interactions such as hydrogen bonding, electrostatic, and hydrophobic interactions [30]. According to [31], it is possible to say that, since both the template and the network are neutral, the chemical interaction between tetracycline and MIP is based on π–π stacking or hydrophobic interactions. If the interaction between the template and the network was mainly based on ionic interactions at that point, there should be a decrease in the binding capacity in acidic and basic conditions due to the repulsion between the same charged form of the tetracycline and the network. Thus, the interaction between tetracycline and the polymeric network is based on mainly π–π stacking or hydrophobic interactions. 

The MIP polymeric film is formed by combining a template, a crosslinker, and a monomer in which a template molecule is used to generate a specific cavity after its removal [32]. The cavity formed in the polymer matrix is complementary in shape, size, and orientation of the chemical functional group of the target analyte to selectively bind it [33]. The electron transfer between the electrode surface and the electrolyte solution is facilitated through these cavities. The electron transfer can also be improved by functionalizing the electrode with nanomaterials such as gold nanoparticles (AuNPs), carbon nanotubes, and other nanoparticles. Among these, AuNP has drawn widespread attention due to its unique properties such as a high surface area to volume, good electrocatalytic activity, and excellent electric conductivity [34]. Therefore the sensor response could be improved by increasing the number of cavities on the MIP electrodes [28]. The development of MIP-based sensor allows rapid, simple, selective, and sensitive detection for TC from animal-origin foods to obtain an enhanced performance due to the fact that it provides an efficient specific recognition site [35].

In the broader field of sensors, many studies have been carried out on the selective binding of TC residues using MIPs. Jiang et al. [8] demonstrated an MIP-based microtiter chemiluminescence sensor for the determination of TC from milk. The authors used minocycline as the template to synthesize the MIP, whose particles were coated in the wells of a conventional 96-well microplate to realize ELISA-based biosensors with a limit of detection (LOD) of 0.5 to 2.0 µM. In this sensor, the absorbed analytes were initiated directly using the highly sensitive bis(2,4,6-trichlorophenyl) oxalate-hydrogen peroxide–imidazole system to emit light. As indicated in the paper, chemiluminescence sensors were simple, rapid, specific, recyclable, and suitable for screening a large number of samples, but their sensitivities were low. Recently, Devkota et al. [14] developed an Au-NP-coated MIP sensor for the electrochemical determination of TC. The authors employed commercial screen-printed carbon electrodes (SPCE) modified with molecularly imprinted overoxidized polypyrrole (MIOPPy), which were modified with Au-NP, obtaining an LOD of 0.65 µM. The overoxidation of polypyrrole, moreover, introduces carboxylic, carbonylic, and hydroxylic groups into the polymeric backbone, which enhances its affinity and specificity to the target molecule. However, MIOPPy usually has negative effects on the electrical conductivity and, consequently, the sensitivity of the electrode, as stated in the paper. Clarindo et al. [36] developed a graphite–polyurethane composite electrode (GPUE) that was modified with methacrylate MIP to detect tetracycline. The goal was to increase the sensitivity and achieve an LOD of 0.555 µM. Density functional theory was also used to evaluate the binding affinity selectivity of the template structure examining the main intermolecular interactions with the monomers. These abovementioned sensors use commercial electrodes, on which they developed different MIPs. The sensor in this work is focused on a laboratory-fabricated nonmetallic conductive electrode, flexible and label-free LIG-based sensor for the determination of TC.

In this work, an alternative approach to achieve the direct electron transfer from the recognition cavities of MIP to the electrode surface is proposed for the indirect detection of TC. The CO_2_ laser-induced graphene (LIG) electrodes were fabricated onto a polyimide (PI, Kapton) flexible substrate, which has the advantages of being cheap and conductive compared to other printed metallic electrodes. Graphene is used as a nonmetallic conductive electrode. According to [37], graphene has already demonstrated its superiority to the well-established carbon nanotube in terms of electrocatalytic activity and macroscopic scale conductivity, thereby suggesting its potential to excel in other areas. For electrochemical sensors, graphene is an ideal material due to its large electrochemical potential window (~2.5 V in 0.1 mM phosphate-buffered saline), and graphene-based electrochemical sensors have superior performance to carbon nanotubes, due to the presence of more *sp*^2^-like planes and edge defects. In addition to this, graphene has an extremely high surface-to-volume ratio, which is theoretically 2600 m^2^·g^−1^, thereby providing a large area for sensing applications. In addition, graphene offers extremely high carrier mobility and high carrier density, and it has low intrinsic noise, thereby providing a high signal-to-noise ratio for better detection. The electrodeposited AuNPs on the LIG electrode were used as a highly conductive and nanostructured imprinted inner layer [38], on which the MIP was directly electropolymerized, acting as a recognition element. In this work, morphology characterization, electrochemical characterization, detection of TC in three different media (buffer, meat, and milk), and stability of the sensor were investigated. The fabricated sensor can be established in the sensor market as an alternative screening tool to detect TC.

## 2. Materials and Methods

### 2.1. Chemical Reagents and Equipment

Chemical Reagents: All reagents used in this work were of analytical grade. TC, gold nanoparticles (AuNPs), *o*-phenylenediamine (oPD), potassium ferricyanide (III) (K_3_[(Fe(CN)_6_]), potassium ferricyanide (II) trihydrate (K_4_[(Fe(CN)_6_]x3H_2_O), potassium chloride, sodium acetate, acetic acid, and phosphate-buffered saline were purchased from Sigma Aldrich (Munich, Germany). Milli-Q water was used to prepare all chemical solutions and cleaning of the electrode at every step. Polyimide (PI) Kapton^®^ type HN with 125 μm thickness (Dupont Corporation, Wilmington, DE, USA) was used as a substrate for the fabrication of the CO_2_ laser-engraved electrodes, due to its thermal stability and resistance to chemical solvents. 

Equipment: A CO_2_ laser engraving and cutting machine (8015 Rayjet-50, Trotec Laser, S.L.U., Barcelona, Spain) with adjustable power and speed was used to fabricate LIG based electrode. A VersaStat 4 potentiostat galvanostat (Princeton applied research, Ametek scientific instruments, Oak Ridge, TN, USA) connected to a personal computer and configured with versastudio software was used for electroanalytical measurements such as cyclic voltammetry (CV), chronoamperometry, and differential pulse voltammetry (DPV). Scanning electron microscopy (SEM) (NVision40 from Carl Zeiss, Oberkochen, Germany), Raman spectroscopy (NRS-5100 dispersive micro-Raman spectrometer from JASCO International Co. Ltd., Tokyo, Japan), and X-ray photoelectron spectroscopy (XPS) (Kratos Analytical Ltd., Manchester, UK) were used to characterize surface morphology, defects on the structure of the graphene material, and surface chemical composition and atomic ratio characterization, respectively. 

### 2.2. CO*_2_* LIG Electrode Fabrication

The electrodes were fabricated following the laser-scribing technique to obtain LIG electrodes on a PI (Kapton, Dupont Corporation, Wilmington, DE, USA) substrate. For that, we employed a continuous CO_2_ scribing with a laser wavelength of 10.6 µm [39]. The ablation process was optimized in order to find the best combination of both laser power and speed to achieve a good conductivity (i.e., a low sheet resistance). This optimization was made using the line transmission method (LTM) with different laser powers (from 4.5 W up to 9 W in steps of 0.75 W) at different speeds (10, 15, and 20 cm/s), as described in the Supplementary Materials (Figure S2A,B).

### 2.3. Surface Morphology Characterization

The formation of LIG and its morphology were characterized by SEM, micro-Raman spectroscopy, and XPS. SEM images were taken at 5 kV extraction and acceleration voltage. Raman spectroscopy analysis was carried out using a dispersive micro-Raman spectrometer (JASCO NRS-5100, Easton, PA, USA) with a green diode (Elforlight G4-30; Nd:YAG, λ = 532 nm, data interval: 1 cm^−1^, exposure time: 15 s, accumulations: 5, center number: 1469.99 cm^−1^) as an excitation source to characterize the formation of the graphene-derived material. The XPS experiments were carried out on a Kratos Axis Ultra-DLD (Manchester, UK), using an X-ray (Al Kα, hv = 1486.6 eV) power of 450 W in a vacuum chamber, where the pressure was kept below 10^−^^10^ Torr, with a scanned area of 300 μm × 700 μm, pass energy of 40 eV, sampling depth of 10 nm, and step of 1 eV, which was used to determine atomic percentage of carbon and oxygen and the presence of their functional groups.

### 2.4. CO*_2_* LIG-Based MIP Sensor Fabrication Process

The stepwise sensor fabrication process is indicated in the Supporting Material (Figure S1). A three-electrode LIG system was fabricated using the optimum configuration of the CO_2_ laser onto the flexible PI substrate. The manufactured sensor consists of a working (D = 4 mm), counter, and a reference electrode. To maintain a stable potential during electrochemical measurement, the reference electrode was glued with a silver/silver chloride ink [40]. After engraving the PI, all the electrochemical processes (electrode cleaning, AuNP electrodeposition, MIP electropolymerization, and rebinding of the template) were optimized as described in separate sections. In general, the CO_2_ LIG sensor fabrication process is indicated in Supplementary Materials (Figure S1). 

**Figure 1:**
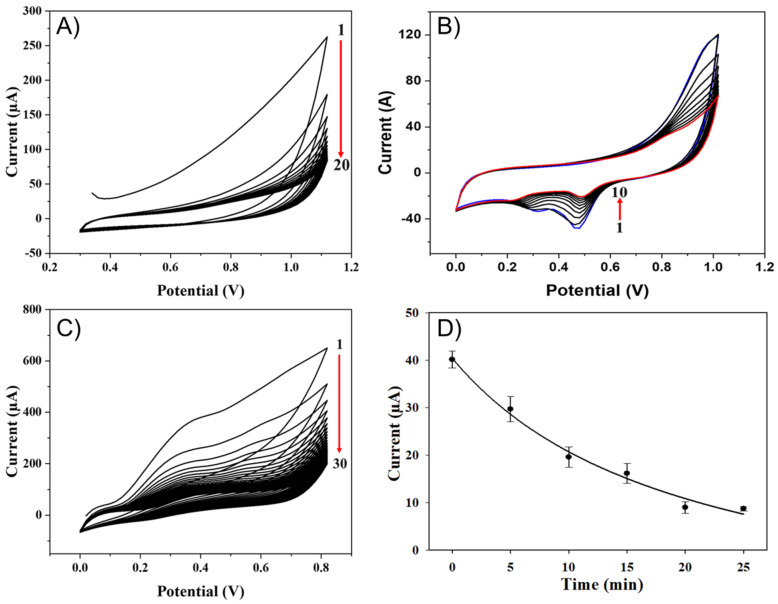
Optimization of the sensor fabrication steps: (**A**) for electrode cleaning, cyclic voltammetry curves showing that the increasing number of cyclic voltammetry cycles (up to 20) leads to a stable curve; (**B**) cyclic voltammetry curves for electrodeposition of AuNPs, where the blue and red-colored curves show before and after electrodeposition of AuNPs, respectively; (**C**) electropolymerization of MIP, where cyclic voltammetry curves show that the increasing number of cycles (up to 30 cycles) leads to a stable curve; (**D**) the rebounding of the tetracycline on the cavities takes up to 25 min until the saturation of the cavities.

Cleaning of the electrode: After fabrication, the LIG electrodes were electrochemically cleaned to remove extraneous materials. Cleaning was carried out by performing CV at a potential sweeping of +0.3 to +1.0 V with a scan rate of 100 mV/s in acetate buffer (pH 5.0, 25 °C) until a stable CV curve was obtained [41]. The typical current generated during CV measurement was decreased with an increasing number of cycles until it became stable after 20 cycles as indicated in Figure 1A. The decrease in the current generation is due to the removal of some superficial particles of graphene and impurities that were not strongly bound to the surface of the LIG electrode [42]. Finally, the electrodes were rinsed with Milli-Q water and air-dried at room temperature (25 ± 2 °C).

Electrodeposition of AuNPs: According to [43], Au nanoparticles can be synthesized from hydrogen tetrachloroaurate (HAuCl_4_). However, the AuNPs used in this work were purchased from Sigma-Aldrich with a diameter of 15 nm. After electrochemical cleaning of the LIG electrodes, 20 µL of 0.1 nM AuNPs (15 nm diameter) and 50 µL of PBS buffer (pH = 7.4 at 25 °C) containing 0.1 M KCl as an electrolyte solution were drop-casted on top of the sensing area of the sensor. The electrodeposition of AuNPs was performed by CV at a sweep potential of 0.0 to +1.0 V and a scan rate of 100 mV/s. The LIG-AuNPs modified layer was used as a highly conductive and nanostructured electrochemically imprinted inner layer [38]. As shown in Figure 1B, stable CV curves were obtained after 10 cycles indicating the generation of a stable current. The reduction peak around 0.5 V shows the reduction of gold (Au) (Figure 1B) and its attachment to the surface of the electrode as indicated in the studies [43,44]. Figure 1B indicates the sensor performance before (blue-colored curve) and after (red-colored curve) optimization of AuNP electrodeposition.

Electropolymerization of MIP: For MIP fabrication, 0.09 M oPD in acetate buffer solution (pH = 5.0 at 25 °C) and 0.03 M TC in methanol were prepared and mixed 1:1, *v*/*v* ratio [41], and 20 µL of this mixture and 50 µL of acetate buffer solution (pH = 5.0, 25 °C) were drop-casted on top of the sensing area of the sensor. The CV was used for electropolymerization of MIP with the potential range between 0.0 and +0.8 V, at 100 mV/s, until a steady curve was obtained. This step is very important in the development of MIP-based sensors because it contains the template that will be removed to form the cavities to rebound the target analyte during testing the sensor. Here, optimization of the CV cycles to obtain a complete polymerization is important. In this regard, the stable curve was obtained at 30 CV cycles of electropolymerization as indicated in Figure 1C. The current generated decreased when the number of CV cycles was increased. The highest current was generated during the first CV scan and, when it reached the optimum (30 cycles), the oxidation peak current density became smaller, and the current generated become stable. This is because the oPD fully interacted with TC and formed a polymeric film on the surface of the electrode. The irreversible peak obtained around 0.35 V indicates the oxidation of oPD [41], and no reduction peak was observed. The results confirmed the development of a TC–oPD polymer film on the surface of the AuNP-modified LIG electrode. The oxidation peak obtained was in line with the work of Li et al. [41].

Template removal: After MIP electropolymerization, imprinted TC molecules should be removed from the surface of the polymeric membrane of the MIP modified electrodes to form a stereo cavity. For this purpose, the MIP electropolymerized electrodes were soaked in methanol/acetic acid (1:1, *v/v*) solution overnight and then rinsed with Milli-Q water. 

Optimization of TC rebounding time: The rebounding of the TC was performed by drop-casting a TC solution on the electrode and waiting for a different set of times (0 to 25 min) in which the TC was rebounding to the cavities at room temperature (25 ± 2 °C). After rebounding, the electrodes were washed with Milli-Q water to remove unbound TC from the surface of the electrode. The electrochemical technique used to understand the best rebounding time was chronoamperometry, working at a supplied potential of +0.9 V using 0.01 M [Fe(CN)_6_]^3−/4−^ containing 0.1 M KCl as an electrolyte solution. The results of the rebounding are shown in Figure 1D. As shown in the figure, the generated current decreased with increasing incubation time. This is because, when the incubation time was small, fewer TC molecules were bound to the cavities formed and, as a result, the contact between the electrolyte solution and the electrode surface was high enough to facilitate the electron transfer. However, when the incubation time was high, more TC molecules had the chance to rebound to the cavities and reduce the contact between the electrolyte solution and the electrode surface that prevents the electron transfer. As shown in the graph, after 20 to 25 min of bounding time, the generated current remained constant. This is because all the free cavities were occupied with the TC molecules, which blocked the electron transfer. Hence, in this study, 25 min was taken as an optimum rebounding time. 

### 2.5. Electroanalytical Measurement

DPV was used as an electrochemical technique to test the performance of the LIG-based MIP sensor for TC detection and to obtain the calibration curve. DPV was performed in a potential range of −0.4 V to +0.8 V with 4 mV potential step height, 50 mV/s scan rate, and 50 mV pulse height using 0.01 M [Fe(CN)_6_]^3−/4−^ containing 0.1 M KCl solution as the supporting electrolyte solution at room temperature (25 ± 2 °C). Here, 20 μL of different TC concentration solutions were drop-casted on the sensing area, waiting for the incubation time (25 min). During this waiting time, the TC rebounded to the cavities in the MIP. Afterward, the electrodes were rinsed with Milli Q water to remove the unbound TC from the surface of the sensor. Finally, electrochemical DPV measurement was performed. 

### 2.6. Preparation of Milk and Meat Samples

Milk and meat samples were purchased from a local market in Bolzano, Italy. The milk sample (50 mL) was centrifuged at 10,000 rpm for 15 min at 4 °C to separate the fat globules. Afterward, defatted milk samples were collected and used for further analysis. The lean meat sample was chopped into small pieces and then squeezed to obtain meat extract. This extracted juice was centrifuged at 10,000 rpm for 15 min at 4 °C to separate the solid particles. Finally, the TC-free defatted milk and the meat extract were spiked with different concentrations of TC to test the analytical performance of the developed sensor in a real sample [8,14]. 

## 3. Results and Discussion 

### 3.1. Optimization of CO*_2_* Laser Power and Speed for Electrode Fabrication

**Figure 2:**
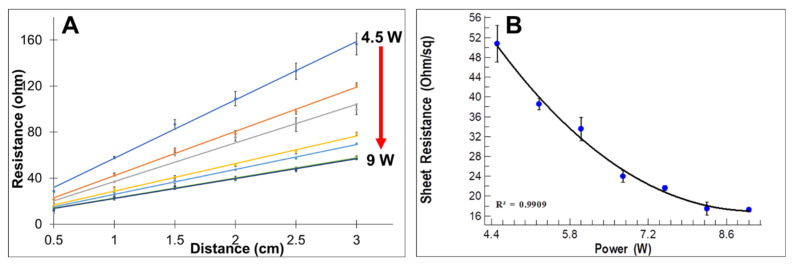
Optimization of electrode fabrication parameters for 10 cm/s CO_2_ speed: (**A**) resistance of LIG electrodes fabricated with different CO_2_ powers; (**B**) respective sheet resistance.

All powers (4.5–9 W) with 0.75 W difference and three different speeds (10, 15, and 20 cm/s) were used to scribe the PI. Below 4.5 W for 10 and 15 cm/s speeds and below 6 W for 20 cm/s speed, the LIG was not well formed, as shown in Table S1. The time elapsed to engrave the 1 cm wide and 12 cm long stripe was 27.33 min, 16.48 min, and 13.17 min for 10 cm/s, 15 cm/s, and 20 cm/s, respectively. The line transmission method (LTM) was used to extract the sheet resistance of the electrodes [45,46,47,48,49]. First, the resistance was measured with respect to the distance and then converted to sheet resistance. Table S1 shows a summary of the sheet resistance of LIG. At each engraving speed, the sheet resistance decreased with increasing power. For the same engraving power, the sheet resistance increased with increasing engraving speed. Therefore, engraving with low speed and applying high power results in the formation of more layered graphene due to the change in carbon atom hybridization from *sp*^3^ to *sp*^2^ [50]; as a result, the conductivity was improved, as indicated by the lower sheet resistance values obtained. In this case, the sheet resistance of 17.44 Ω/sq. and 17.23 Ω/sq. approached stability between 8.25 W and 9 W for 10 cm/s speed, respectively. The laser power of 8.6 W and 10 cm/s speed was taken as an optimum parameter for subsequent electrode fabrication. Finally, stripes were fabricated with the optimum parameters, and the resistance was measured (Figure 2A) and converted to the sheet resistance (Figure 2B). The measured sheet resistance was 17.274 Ω/sq. For the remaining experiments, the three-electrode system electrodes were engraved using these optimum values of CO_2_ laser power and speed. The sheet resistance values for 15 cm/s and 20 cm/s speeds are shown in Figures S3A,B and S4A,B, respectively.

### 3.2. Characterization of the Electrode

**Figure 3:**
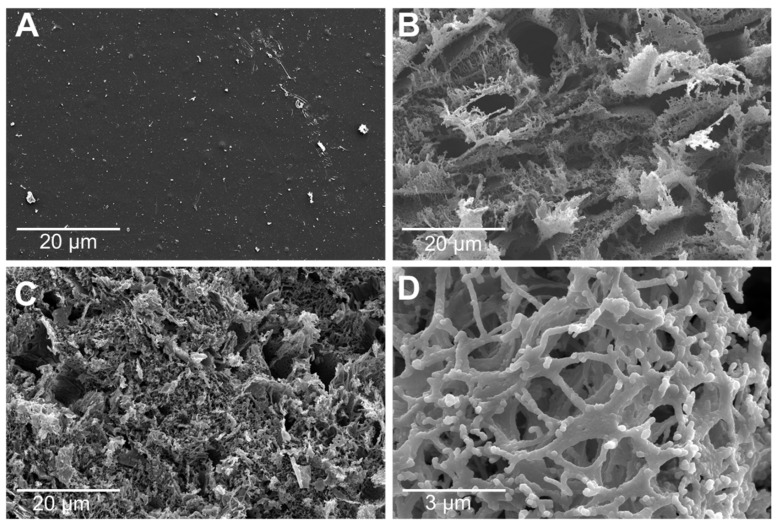
SEM images at 5 kV extraction and acceleration voltage, and at 7.2 mm working distance with scale bar of 20 µm, for (**A**) bare PI, (**B**) LIG electrode fabricated with 10 cm/s speed and 4.5 W power, and (**C**) LIG electrode fabricated with 10 cm/s speed and 8.6 W power. (**D**) High resolution at 3 µm showing the graphite network.

Surface morphology characterization: SEM was used to characterize the surface morphology of the LIG electrode. The SEM images of PI surface and LIG engraved at 4.5 W and 8.6 W laser power and 10 cm/s laser speed are shown in Figure 3 for unengraved PI (Figure 3A) and engraved LIG at 4.5 W (Figure 3B) and 8.6 W (Figure 3C). The surface of the engraved LIG exhibited a porous foam-like structure due to the extreme increase (91.24%) in the atomic percentage of carbon [51]. Compared to the PI, LIG showed a porous microstructure surface that resulted in a high surface area. The flakes of LIG showed a continuous network that exhibited a relatively low sheet resistance. Figure 3D shows the typical network structure and multilayer nature, which indicates that the entire body of PI was converted to graphitic structures through laser engraving. Actually, from the SEM images, it is impossible to distinguish that it is graphite; rather, we can only analyze the morphology of nanostructures. For convincing proof, it was necessary to obtain Raman spectra to validate the formation of graphite and XPS spectra to determine the elemental content.

**Figure 4:**
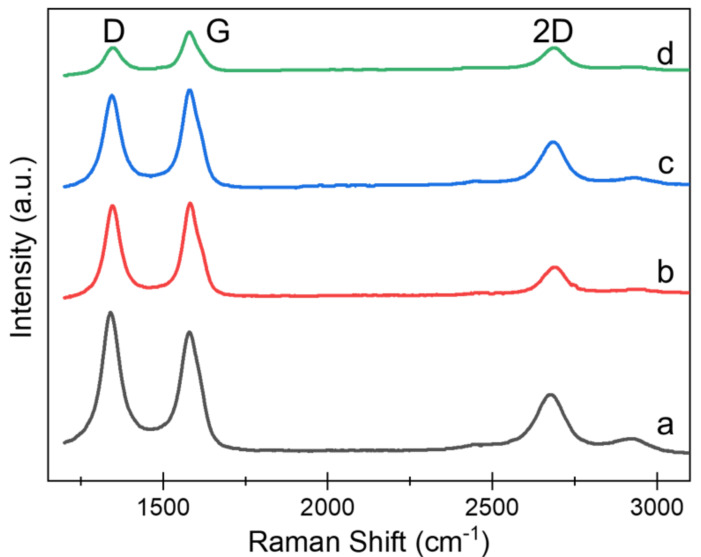
Raman spectra for different laser powers at a speed of 10 cm/s: (**a**) 4.5 W, (**b**) 6.0 W, (**c**) 8.6 W, and (**d**) 9.0 W.

Raman spectroscopy analysis: A dispersive micro-Raman spectrometer with an excitation source with a wavelength of 532 nm at room temperature was used for Raman spectroscopy analysis. The spectra obtained were used to determine the nature of the CO_2_ LIG engraved PI. Raman spectra (Figure 4) of the LIG were mainly composed of three different peaks (2D, G, and D) which had specific indications on the structure of the LIG. D, G, and 2D peaks were located at ~1345 cm^−1^, ~1580 cm^−1^, and ~2700 cm^−1^, respectively. These values were in accordance with the work of Lin et al. [52]. It was confirmed from the Raman spectra that the characteristic 2D peak at ∼2700 cm^−1^ of the fabricated material is indeed graphene, as indicated in other similar studies [50,51,52,53,54], which also indicates the multilayer nature of LIG originating from second-order zone-boundary phonons [47]. The *sp*^2^-hybridized carbon network of the graphitic material is associated with the G peak, while the D peak denotes the presence of bent graphene or defects in the graphene structure [55].

It was observed that the G peak increased and the D peak decreased with the increasing engraving power. This may be due to the increase in LIG layer formation, the change of carbon atoms from *sp*^3^ to *sp*^2^ hybridization, and a partial restoration of the hexagonal honeycomb crystal lattice of graphene [39]. The increase in the intensity of D peak indicated that a high-power laser can generate a more defective and disordered structure [56], as indicated in the graph.

Table 1 shows the ratios of the peaks with respect to the engraving power. The intensity ratio of D to G peaks (I_D_/I_G_) with an approximate value of 1 depicts that the surface of the PI was derived to graphene [53] and decreased with the increase in engraving power, which can be attributed to the graphitization degree of the PI film which is positively related to engraving temperature [57]. On the other hand, a relatively smaller value of the intensity ratio of 2D to G peak (I_2D_/I_G_) ratio indicates the bending of the graphene sheet [53]. The information of the D, G, and 2D peaks from Raman spectroscopy listed in Table 1 verifies the formation of the characteristics of multilayer graphene with laser engraving of PI sheets [57].

The development of the I_d_/I_g_ and I_2d_/I_g_ ratios was plotted as a graph (Supplementary Figure S5). As observed in Figure S5, the I_D_/I_G_ ratio slightly decreased with increasing engraving power, but its value of ~1 confirmed the crystalline nature of the ablated surface, whereas the increase in I_2D_/I_G_ ratio indicated a reduction in the number of graphene layers.

X-ray photoelectron spectroscopy (XPS) analysis: The XPS analysis was performed at a pressure of 10^−10^ Torr and an X-ray power of 450 W in a vacuum chamber. This analysis can help to extract the atomic percentage and the identification of carbon–oxygen compound changes [58]. The total spectra for the chemical compositions are depicted in Figure S6A, while Figure S6B,C show the zoomed-in view of carbon spectra before and after the laser engraving process, respectively. As indicated in Figure S6A, the XPS spectra for C, N, and O peaks were at ~284, ~401, and ~530 eV binding energies, respectively, in accordance with the work of Mu et al. [59] and the engraved surfaces showed the domination of carbon and oxygen atoms with a very high-C spectrum. In this case, engraving increased the C–O functional groups. On the other hand, the N peak became diminished. 

XPS spectra of carbon 1*s* with high resolution were studied in detail to demonstrate the nature of the changes of bonding formed, as indicated in Figure S6B,C. The intensity of engraved C–C peaks was around threefold higher than that of the PI substrate. This may be due to the higher isolation of *sp*^2^ hybridized atoms caused by the CO_2_ laser. These peak curve fits were used to estimate the positions of the peak for the functional group and to predict atomic concentrations. The most prominent peak belonged to the carbon backbone and was observed around 284.6 eV with an atomic concentration of 91.24%. The carbon bonds for C–O, O–C–O, and O–C=O were obtained around 286.7 eV, 288.0 eV, and 288.5 eV, respectively. The peak at 284.5 eV was assigned to carbon atoms with *sp*^2^ hybridized orbitals and was asymmetric due to defragments. This peak represents the π* state of C–C (π* excitations). It is assumed that the chemical groups with *sp*^2^ hybridized orbitals such as epoxy groups were formed at initial stages. and then the chemical groups with *sp*^3^ hybridized orbitals such as the hydroxyl groups appeared.

The atomic percentages of C, O, and N and the respective C/O and C/N ratios of XPS data for unengraved and engraved materials are indicated in Table S2. The engraved surfaces mainly contained carbon and oxygen elements, along with reduced N, which led to a drastic increase in both ratios.

The increment in C-to-O ratio (13.69) was more than double for the engraved systems compared to PI, while the C-to-N ratio (285.13) was almost 14 times that of PI, which indicates the increment in C and O atoms and reduction in N atoms. The C-to-O and C-to-N ratios increased from 5.83 and 21.48 in PI to 12.33 and 285.13 in engraved systems, respectively. Therefore, N atoms can be released more easily than O atoms, contrary to the case of thermal carbonization.

The ablation process involves significant modifications of the C 1*s* peak intensity and its shape. These results confirm that, as the laser power increased, the number of C=O, C−O−C, and C−N bonds decreased, while the C−C peak increased, which is in agreement with the atomic percentages obtained.

### 3.3. Electrochemical Characterization of LIG Electrode after Each Modification Step

**Figure 5:**
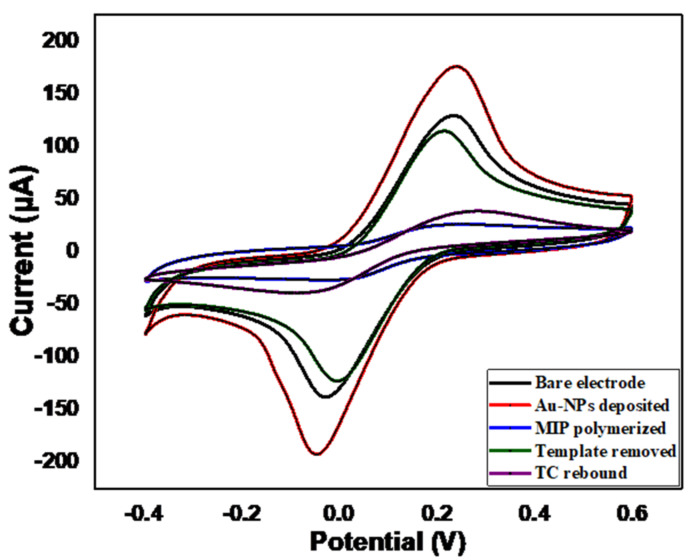
Cyclic voltammetry curves obtained after each modification step of LIG electrode.

CV was used to characterize the stepwise modification of the LIG electrode using 0.01 M [Fe (CN)_6_]^3−/4−^ that contains 0.1 M KCl solution in a potential range of −0.4 to +0.8 V at a scan rate of 100 mV/s. The results are shown in Figure 5. First, CV was performed on the bare LIG electrode, and the reduction and oxidation peaks of [Fe(CN)_6_]^3−/4−^ were observed (black-colored curve). When the AuNPs were electrodeposited (red-colored curve), both peaks (reduction and oxidation) increased dramatically, due to the higher conductivity and high surface area provided by the AuNPs, indicating an enhancement in the electron transfer. After MIP electropolymerization (blue-colored curve), the redox peaks dramatically decreased, which may have been due to the interaction of oPD and the template TC that can cover the surface of the working electrode, thereby blocking the electron transfer between the electrode and the electrolyte solution. The dramatic redox peak decrement was due to the poor electrical conductivity of the polymeric film that covers the surface of the electrode and prevents the transfer of electrons between the electrode surface and the electrolyte solution. An increase in the peak current after template (TC) removal (green-colored peak) indicates a successful removal of the template and, hence, a better electron transfer in comparison with the MIP modified electrode. On the other hand, the redox peaks were slightly lower compared to the unmodified electrode, because the electrode was still covered with poly-oPD, whose conductivity is lower than LIG electrode. After TC rebinding, the redox peaks (pink-colored curve) increased compared to the MIP modified electrode. This may be because the cavities formed during template removal were not completely rebound in the TC molecule. The authors of [60] compared the cyclic voltammogram and electrical impedance spectrum (EIS) to characterize the electrochemical behavior of MIP-based electrodes with different modifications and obtained similar trend.

### 3.4. MIP Sensor Performance for TC Detection 

**Figure 6:**
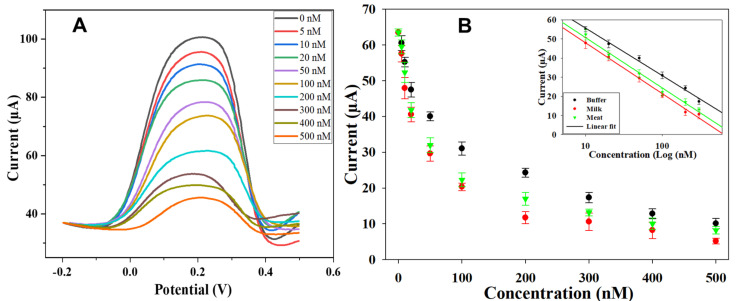
Sensitivity test of the sensor: (**A**) the DPV curves for different TC concentration in acetate buffer; (**B**) the calibration curves extracted from the DPV curves heights (inset: linear range of the sensors).

For the detection of TC, the sensor was fabricated using the optimized conditions stated in Section 3.3. A stock solution (1 mM) of TC was prepared in methanol; afterward, different concentrations of TC (5–500 nM) were prepared by diluting the stock solution in acetate buffer (pH = 5.0). The sensing area was covered with the prepared solution, and DVP was used to obtain the electrochemical signal because it was shown to give an electrical signal with higher resolution and enhanced sensitivity [38]. In previous centuries, the electrochemical reductive behaviors of some TC antibiotics including demeclocycline (DMC) were examined, but there are currently studies indicating its oxidative behavior. The electrochemical behavior of TC was studied using DPV in 0.1 M KCl containing 1.0 mM K_3_[Fe(CN)_6_]. During DPV measurement, the redox current peak heights decreased with the increasing TC concentrations, as seen in Figure 6A. During the rebinding of the TC, when the concentration is low, there are more free cavities; thus, there is more space for electron transfer between the electrolyte solution and the electrode surface. However, when the concentration increases, most of the cavities are filled with the TC molecule, reducing the active space for electron transfer, thereby lowering the peak current. To further test its practical application, the sensor was validated in real samples (milk and meat). For this purpose, different concentrations (5–500 nM) of TC were spiked in defatted milk and meat extract, and their respective DVP curves are presented in Figures S7A and Figure S7B, respectively. The obtained peaks showed a similar trend to that obtained for buffer. 

The calibration curves were realized on the basis of the generated current (DPV) versus the TC concentrations, as indicated in Figure 6B. Each point in the calibration curve represents an average of five measurements (*n* = 5) at each concentration of the TC. The working range for acetate buffer, milk, and meat samples was between 5 to 500 nM as indicated in Figure 6B. We found a linear regression between the logarithm of the TC concentration and the generated current in the range 10 to 300 nM (inset of Figure 6B), as expressed below.

I (μA) = −10.80ln(C) + 80.522 (*R^2^* = 0.992), (1)

I (μA) = −11.55ln(C) + 74.548 (*R^2^* = 0.993), (2)

I (μA) = −11.42ln(C) + 76.990 (*R^2^* = 0.991), (3)

for acetate buffer (Equation (1)), milk (Equation (2)), and meat extract samples (Equation (3)). The limit of detection (LOD) was calculated using the following equation:

LOD = 3δ_b_/m, (4)

where δ_b_ is the standard deviation of the blank measurements (*n* = 5), and m is the slope of the linear regression curve. LOD was calculated as 0.32 nM, 0.85 nM, and 0.80 nM for buffer, milk, and meat extract samples, respectively. The higher LOD for real samples is expected because of food matrix interferences [61]. The remaining fat globules and nonfat solid materials in milk and meat extract may have blocked some cavities and prevented the rebounding of the TC molecule. The fabricated disposable sensors in this work showed good reproducibility over the range of the concentration of 5–500 nM of TC in acetate buffer, defatted milk, and meat extract. For example, at 100 nM TC concentration, the standard deviations (*n* = 5) were 1.80, 1.05, and 1.95 for a buffer, defatted milk, and meat extract samples, respectively. These standard deviations indicate good sensor reproducibility. In recent work, researchers developed MIP-based electrochemical commercial sensors based on metallic electrodes for the determination of TC (Table 2) and obtained an LOD between 1 nM and 0.026 mM.

Ziang et al. [8] developed a microtiter chemiluminescence sensor on a conventional microtiter ELISA plate. The linear range of the developed sensor was in the range of 0.004 to 1000 ng/mL with an LOD of 1 nM for TC. Devkota et al. [14] reported a screen-printed carbon electrode (SPCE)-based MIP sensor using pyrrole as a monomer and TC as a template. The sensor linear range was in the range of 1–20 mM with an LOD of 0.65 mM. Pernites et al. [27] developed a thermally polymerized MIP sensor on Ti substrate electrodeposited with a micro–nano Pt cluster (MIP-Pt/Ti) and obtained a linear range of 0.1–10 mM and an LOD of 0.026 mM. Compared to Ziang et al. [8], our biosensor showed lower sensitivity. This was due to the use of sensitive conventional instruments (ELISA). However, the disposable sensor developed in this work compared to the ELISA method has many advantages in terms of cost, measurement time, and sample preparation. Compared to other printed sensors, our developed sensor in this work showed an improvement in LOD (0.85 nM and 0.80 nM) for TC detection in milk and meat extract samples, respectively. This is an indication of the realization of a fast, cost-effective, nonmetallic sensor for the detection of TC and its application in real samples such as milk and meat.

The repeatability and reproducibility tests were described using the standard deviations of the measurement (*n* = 5). The maximum standard deviations obtained for buffer, meat and milk were 2.02, 2.80, and 2.97, respectively. In addition, the relative standard deviation (%RSD) was calculated as 4.26, 5.35, and 6.20 for buffer, meat, and milk, respectively. According to these values, the deviation was acceptable, and the developed system was reproducible and repeatable.

### 3.5. Time Stability Test

**Figure 7:**
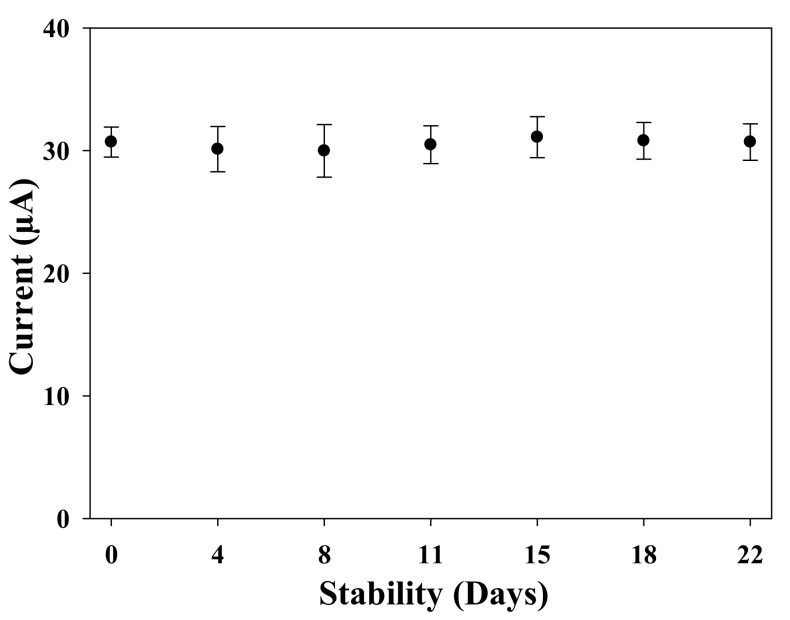
Time stability test of the MIP electrochemical sensors.

The time stability test is a very important parameter to predict the shelf-life of the sensor. For this experiment, the sensors were fabricated in the same conditions and stored dry at 4 °C until use. The experiments were carried out for 22 days to detect 100 nM of TC in the acetate buffer. Each measurement represents an average of five measurements (*n* = 5), and the corresponding results are shown in Figure 7. The results showed that, for the entire test period, the sensor response maintained stability with a variability of 1.3–2.3% compared to the initial response. 

## 4. Conclusions

The MIP-based electrochemical sensor was developed using CO_2_ LIG and then modified with AuNPs to enhance the electrochemical signal; finally, an MIP process was employed as a synthetic recognition element to detect antibiotic residue, TC, in animal-origin food samples. Graphene was used as a nonmetallic conductive electrode, while AuNPs were used as a highly conductive and nanostructured imprinted inner layer to enhance the electron transfer. MIP after the removal of the template formed specific cavities, which were used as a synthetic recognition element to rebind the target analyte. The LOD of the sensor was calculated to be 0.32 nM, 0.85 nM, and 0.80 nM in the buffer, milk, and meat extract samples, respectively, with a working range of 5 nM to 500 nM; an improvement in sensitivity was achieved compared to state-of-the-art sensors in the literature. The sensor presented good analytical performance in terms of LOD, stability over 22 days, and reproducibility. From the obtained results, we can conclude that the developed sensor is a promising analytical tool for TC detection. As a promising alternative sensor, it has the advantages of being cheap, disposable, rapid, and easy to operate; thus, it is suitable to detect TC for a wide range of applications such as environmental monitoring and pharmaceutical analysis. To assess the selectivity and, in particular, to improve the LOD of the analysis, more studies should be carried out. The effects of pH and temperature on the sensing performance could be investigated in the future work.

## Figures and Tables

**Table 1:** Raman spectrogram intensities of the D, G, and 2D peaks and their ratios.

Laser Power (W)	Laser Speed (cm/s)	D Peak	G Peak	2D Peak	I_D_/I_G_	I_2D_/I_G_
4.5	10	400.31	371.27	278.76	1.08	0.71
6.0	10	326.92	330.56	235.89	0.99	0.75
8.6	10	329.29	337.86	260.72	0.97	0.77
9.0	10	222.74	245.72	222.59	0.91	0.91

**Table 2:** Comparison of the LODs of MIP-based different sensors developed so far.

Type of Sensor	Analyte	Sample	Linear Range	LOD	Reference
Microtiter chemiluminescence	Tetracycline	Milk	0.009–2250 nM	0.002 nM	[8]
AuNP-coated screen-printed carbon electrodes	Tetracycline	Shrimp sample	1–20 mM	0.65 mM	[14]
Platinum electrode	Tetracycline	PBS buffer	0.225–20 µM	0.058 µM	[27]
CO_2_ LIG electrode	Tetracycline	Acetate buffer,	10–300 nM	0.32 nM	This work
		milk sample,		0.85 nM	
		meat sample		0.80 nM	

## Supplementary Materials

The following are available online at https://www.mdpi.com/article/10.3390/s22010269/s1: Figure S1. CO_2_ LIG sensor fabrication process; Figure S2. Sheet resistance measurement system using the line transmission method (LTM): (A) resistance measurement with respect to the distance; (B) the calibration curve of resistance vs. each distance (*n* = 3); Figure S3. Sheet resistance measurement for the electrodes fabricated with 15 cm/s speed: (A) resistance measurement of each speed with respect to the distance; (B) sheet resistance extracted from the slop of each resistance measurement; Figure S4. Sheet resistance measurement for the electrodes fabricated with 20 cm/s speed: (A) resistance measurement of each speed with respect to the distance; (B) sheet resistance extracted from the slope of each resistance measurement; Figure S5. XPS graphs of LIG: (A) fabricated with different power; (B) PI and (C) carbon 1*s* peaks at optimum power (8.6 W); Figure S6. DPV curves for different TC concentrations to test the sensitivity of the sensor: (A) in milk samples; (B) in meat extract samples; Table S1. Sheet resistance of LIG fabricated electrodes with different CO_2_ laser speed and power; Table S2. Carbon, oxygen, and nitrogen atomic percentages and their ratio extracted from the XPS spectra for the LIG fabricated electrodes with different engraving power at 10 cm/s speed.
