# Supplementary material for: Laser-Induced Graphene Electrodes Modified with a Molecularly Imprinted Polymer for Detection of Tetracycline in Milk and Meat
Source: Sensors (Basel). 2021 Dec 30;22(1):269. doi: 10.3390/s22010269 (PMC8749683; doi:10.3390/s22010269)
Supplement: Supplementary file 1 [file sensors-22-00269-s001.zip › sensors-1516536-supplementary.pdf]

Supplementary Materials

Laser-Induced Graphene Electrodes Modified with a Molecularly Imprinted Polymer for Detection of Tetracycline in Milk and Meat

Biresaw D. Abera [1,2,*], Inmaculada Ortiz-Gómez [3], Bajramshahe Shkodra [1], Francisco J. Romero [4], Giuseppe Cantarella [1], Luisa Petti [1,*], Alfonso Salinas-Castillo [3], Paolo Lugli [1] and Almudena Rivadeneyra [4]

1 Faculty of Science and Technology, Free University of Bolzano, 39100 Bolzano, Italy; Bajramshahe.Shkodra@natec.unibz.it (B.S.); giuseppe.cantarella@unibz.it (G.C.); paolo.lugli@unibz.it (P.L.)
2 Faculty of Chemical and food Engineering, Bahir Dar institute of technology, Bahir Dar University, 6000 Bahir Dar, Ethiopia
3 Department of Analytical Chemistry, University of Granada, 18071 Granada, Spain; inmaog@ugr.es (I.O.-G.); alfonsos@ugr.es (A.S.-C.)
4 Pervasive Electronics Advanced Research Laboratory (PEARL), Department of Electronics and Computer Technology, University of Granada, 18071 Granada, Spain; franromero@ugr.es (F.J.R.); arivadeneyra@ugr.es (A.R.)
* Correspondence: chembires@gmail.com (B.D.A.); Luisa.Petti@unibz.it (L.P.)

1. CO_2 LIG Electrode Fabrication

The CO_2 LIG electrode fabrication process is indicated in Figure–S1 below.

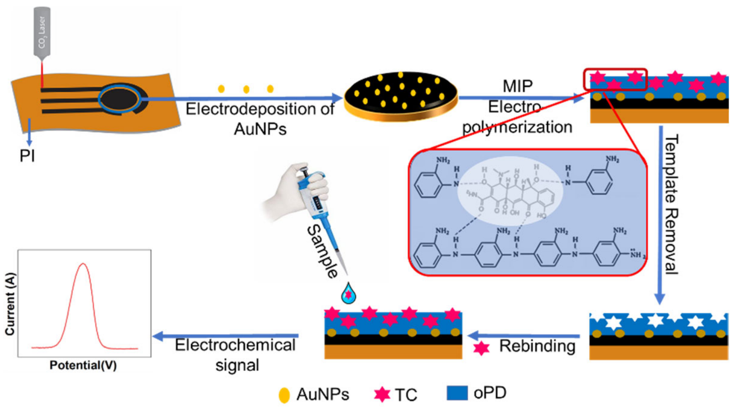

Figure S1. CO2 LIG sensor fabrication process.

2. CO_2 LIG Electrode Fabrication

For the optimization of electrode fabrication parameters, 1 cm wide and 12 cm long stripes were engraved on a PI substrate. To optimize the CO_2 scribing process for electrode fabrication, different laser powers and speeds were studied. A series of laser powers (4.5–9 W) with 0.75 W difference and three different speeds (10, 15, and 20 cm/s) were used to ablate the PI. Then, the electrical sheet resistance (R_{sh}) (Ω/sq.) of the engraved electrodes was extracted using the line transmission method (LTM) [1,2], as shown in Figure–S2.

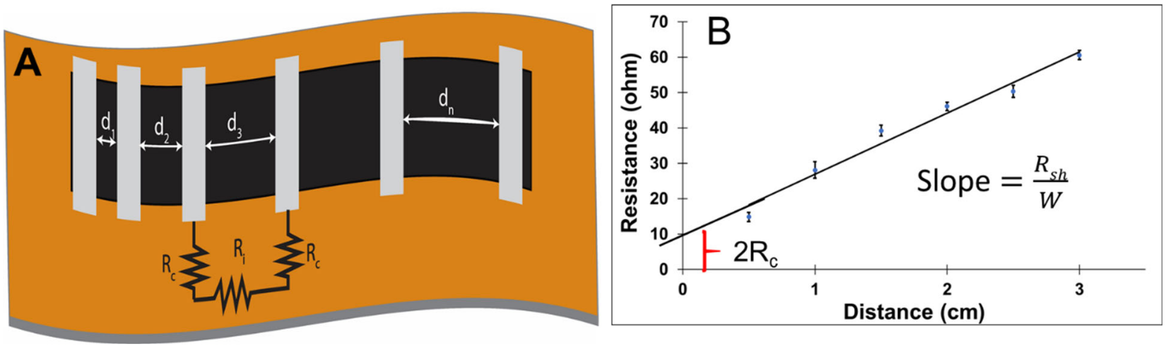

Figure S2. Sheet resistance measurement system using the line transmission method (LTM): (**A**) resistance measurement with respect to the distance; (**B**) the calibration curve of resistance vs. each distance (*n* = 3).

To get R_{sh}, the resistance was measured with respect to the distance (Figure–S2A), and then the calibration curve was plotted as obtained resistance versus its respective distance as indicated in Figure–S2B. The slope of the calibration curve is given by R_{sh} divided by the width of the electrode (W) [3,4]. Then, R_{sh} was extracted from the slope of the calibration curve. R_c indicates the resistance of the surface contact of the electrode which was painted with silver ink.

As indicated in Figure–S3 below, after plotting the calibration curves for each laser-power of electrodes fabricated with 15 cm/s (Figure–S3A), R_{sh} was calculated and plotted for respective laser-power (Figure–S3B).

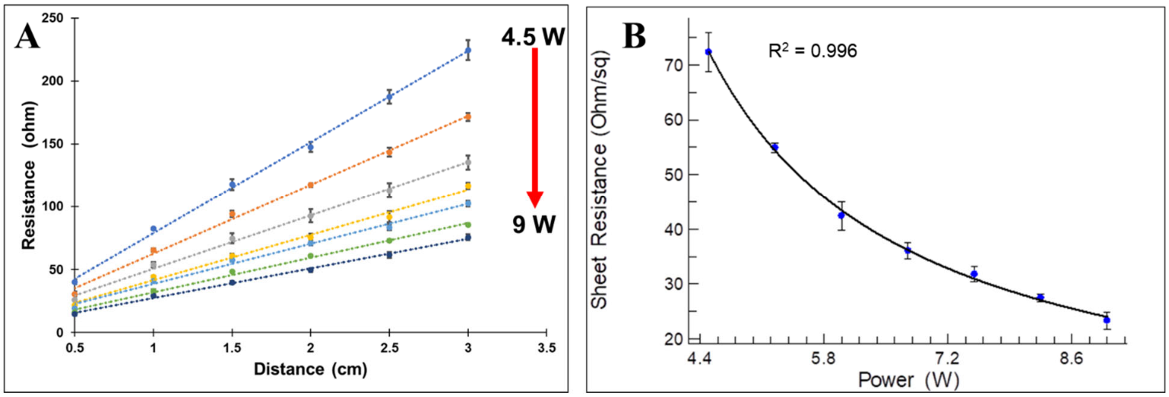

Figure S3. Sheet resistance measurement for the electrodes fabricated with 15 cm/s speed: (**A**) resistance measurement of each speed with respect to the distance; (**B**) sheet resistance extracted from the slope of each resistance measurement.

As indicated in Figure–S4 below, after plotting the calibration curves for each laser-power of electrodes fabricated with 20 cm/s (Figure–S4A), R_{sh} was calculated and plotted for respective laser-power (Figure–S4B).

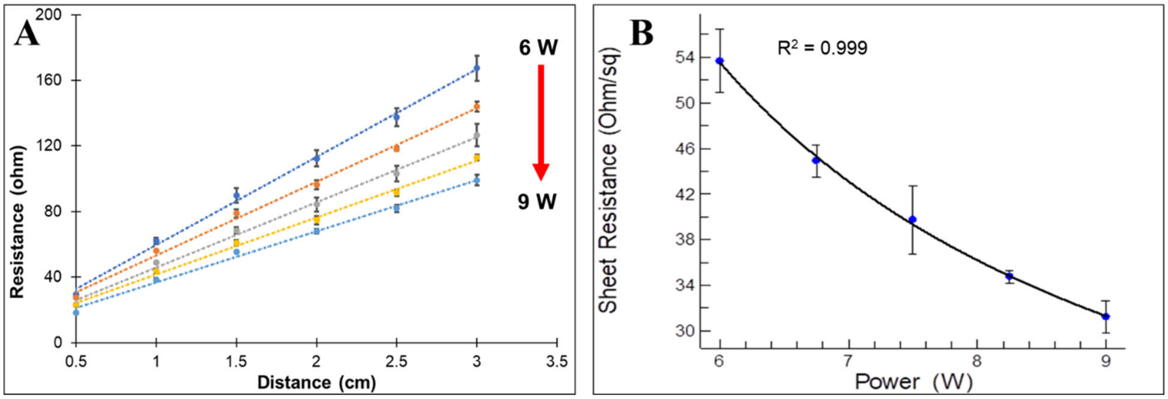

Figure S4. Sheet resistance measurement for the electrodes fabricated with 20 cm/s speed: (**A**) resistance measurement of each speed with respect to the distance; (**B**) sheet resistance extracted from the slope of each resistance measurement.

The R_{sh} of CO_2 LIG electrodes with different engraving laser-power and speed is indicated in Table–S1 below.

Table S1. Sheet resistance of LIG fabricated electrodes with different CO_2 laser speed and power.

Laser Power (W)	Laser Speed (cm/s)	Sheet Resistance (Ω/sq.)
10	4.50	50.76 ± 3.69
	5.25	38.54 ± 1.14
	6.00	33.53 ± 2.35
	6.75	23.97 ± 1.18
	7.50	21.60 ± 0.49
	8.25	17.44 ± 1.30
	9.00	17.23 ± 0.43
	8.60	17.27 ± 1.04
15	4.50	72.34 ± 3.58
	5.25	54.90 ± 0.84
	6.00	42.44 ± 2.61
	6.75	36.07 ± 1.46
	7.50	31.79 ± 1.38
	8.25	27.47 ± 0.65
	9.00	23.55 ± 1.53
20	4.50	--
	5.25	--
	6.00	53.65 ± 2.78
	6.75	44.91 ± 1.39
	7.50	39.75 ± 3.01
	8.25	34.76 ± 0.55
	9.00	31.23 ± 1.41

3. Characterization of the Electrode

The development of the I_d/I_g and I_{2d}/I_g ratios was plotted as a graph (Figure–S5). As observed in Figure–S5, the I_D/I_G ratio slightly decreased with increasing engraving power, but its value of ~1 confirmed the crystalline nature of the ablated surface, whereas the increase in I_{2D}/I_G ratio indicated a reduction in the number of graphene layers.

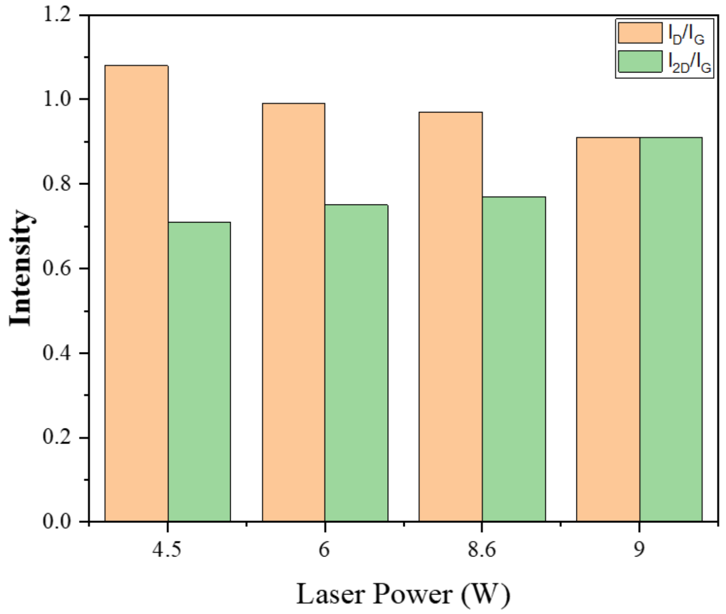

Figure S5. Comparison of intensities at the ratios of D and G peaks.

The total spectra for the chemical compositions are depicted in Figure–S6A, while Figure–S6B,C show the zoomed-in view of carbon spectra before and after the laser engraving process, respectively.

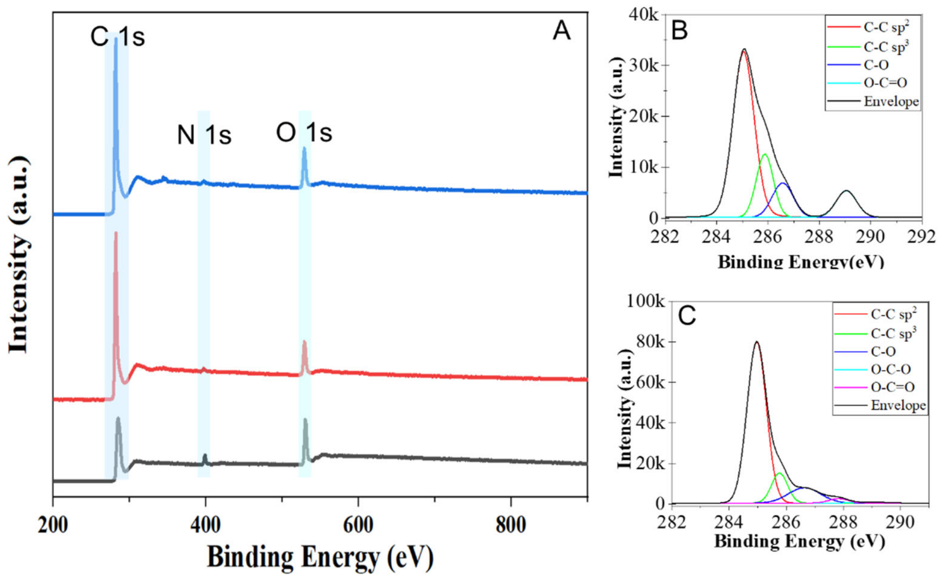

Figure S6. XPS graphs of LIG: (**A**) fabricated with different power; (**B**) PI and (**C**) carbon 1*s* peaks at optimum power (8.6 W).

The atomic percentages of C, O, and N and the respective C/O and C/N ratios of XPS data for unengraved and engraved materials are indicated in Table–S2. The engraved surfaces mainly contained carbon and oxygen elements, along with reduced N, which led to a drastic increase in both ratios.

Table S2. Carbon, oxygen, and nitrogen atomic percentages and their ratio extracted from the XPS spectra for the LIG fabricated electrodes with different engraving power at 10 cm/s speed.

Laser Power (W)	Atomic Percentage (%)			Atomic Ratio	
	C	O	N	C/O	C/N
4.5	92.45	6.75	0.66	13.69	140.08
6.0	91.89	7.15	0.73	12.85	125.88
8.6	91.24	7.40	0.32	12.33	285.13
9.0	90.09	7.31	0.60	12.32	150.15
PI	81.63	13.99	3.80	5.83	21.48

4. MIP Sensor Performance for TC Detection in Milk and Meat Samples

The fabricated sensor was validated in real samples (milk and meat). For this purpose, different concentrations (5–500 nM) of TC were spiked in defatted milk and meat extract, and their respective DVP curves are presented in Figures–S7 in which Figures–S7A if for defatted milk and Figures–S7B is for meat extract.

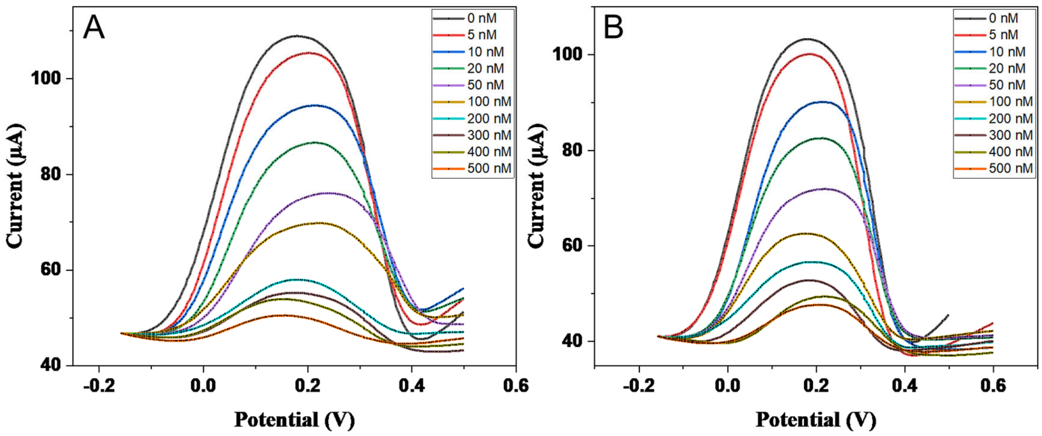

Figure S7. DPV curves for different TC concentrations to test the sensitivity of the sensor: (**A**) in milk samples; (**B**) in meat extract samples.

References

1. Jones, J. Design features of the Murray Hydroelectric Project. *IEEE Trans. Energy Convers.* **1989**, *4*, 160–165, doi:10.1109/60.17906.
2. Lin, J.; Peng, Z.; Liu, Y.; Ruiz-Zepeda, F.; Ye, R.; Samuel, E.L.G.; Yacaman, M.J.; Yakobson, B.I.; Tour, J.M. Laser-induced porous graphene films from commercial polymers. *Nat. Commun.* **2014**, *5*, 5714, doi:10.1038/ncomms6714.
3. Slewa, L. Transmission line method (TLM) measurement of (metal/ZnS) contact resistance. *Int. J. Nanoelectron. Mater.* **2015**, *8*, 111–120.
4. Watanabe, E.; Conwill, A.; Tsuya, D.; Koide, Y. Low contact resistance metals for graphene based devices. *Diam. Relat. Mater.* **2012**, *24*, 171–174, doi:10.1016/j.diamond.2012.01.019.
